# Systemic Corticosteroids in Pediatric Community-Acquired Pneumonia: Evidence, Clinical Outcomes, and Phenotype-Based Therapeutic Considerations

**DOI:** 10.3390/ph19081253

**Published:** 2026-08-09

**Authors:** Marco Masetti, Giulia Zambelli, Sonia Rasmi, Elena Rossini, Salvatore Davide Tramonti, Mandy Ferrocino, Valentina Fainardi, Susanna Esposito

**Affiliations:** Pediatric Clinic, Department of Medicine and Surgery, University Hospital of Parma, 43126 Parma, Italy; marco.masetti@unipr.it (M.M.); giulia.zambelli@unipr.it (G.Z.); sonia.rasmi@unipr.it (S.R.); elena.rossini@unipr.it (E.R.); salvatoredavide.tramonti@unipr.it (S.D.T.); mandy.ferrocino@unipr.it (M.F.);

**Keywords:** pediatric pneumonia, community-acquired pneumonia, corticosteroids, mycoplasma pneumoniae, adjunctive therapy, severe pneumonia

## Abstract

**Background**: Community-acquired pneumonia (CAP) remains a leading cause of pediatric morbidity and mortality worldwide, particularly in low-resource settings. Although antimicrobial therapy is the cornerstone of treatment, disease severity is often influenced not only by pathogen burden but also by dysregulated host inflammation. Systemic corticosteroids have therefore been investigated as adjunctive agents to reduce inflammation-mediated lung injury, but their role in children remains controversial. **Methods**: This narrative review summarizes current evidence on systemic corticosteroid use in pediatric pneumonia. The review focuses on biological mechanisms, clinical indications, patient selection, therapeutic regimens, timing and duration of therapy, clinical outcomes, and safety. Particular attention is given to severe CAP, *Mycoplasma pneumoniae* pneumonia, complicated pneumonia with parapneumonic effusion or empyema, viral pneumonia, wheezing-associated phenotypes, and immunocompromised children. **Results**: Available evidence suggests that corticosteroids may provide benefit in selected pediatric phenotypes characterized by severe inflammation, respiratory compromise, refractory disease, or airway hyperreactivity. Reported benefits include faster fever resolution, reduction in inflammatory markers, shorter hospital stay, more rapid clinical stabilization, and improved radiographic recovery, particularly in severe or refractory *Mycoplasma pneumoniae* pneumonia and selected complicated cases. However, findings are inconsistent, and treatment effects appear strongly dependent on etiology, disease severity, timing of administration, corticosteroid regimen, and patient phenotype. Routine use in uncomplicated CAP or unselected pediatric populations is not supported. Potential adverse effects include hyperglycemia, gastrointestinal and neurobehavioral symptoms, sleep disturbance, immunosuppression with an increased risk of secondary infection, delayed viral clearance in selected viral infections, and possible masking of inadequate anti-infective response or evolving complications. **Conclusions**: Current evidence supports a cautious, individualized, phenotype-driven approach to corticosteroid therapy in pediatric CAP. Well-designed pediatric randomized controlled trials are needed to define precise indications, optimal regimens, timing, and long-term safety.

## 1. Introduction

Pneumonia remains the leading infectious cause of death among children worldwide and continues to represent a major global public health challenge [1,2]. Despite substantial progress in vaccination, antimicrobial treatment, nutritional interventions, and access to healthcare, community-acquired pneumonia (CAP) remains responsible for a considerable burden of pediatric morbidity and mortality, particularly in low- and middle-income countries [1,3]. According to the World Health Organization (WHO), pneumonia accounted for approximately 14% of all deaths among children younger than five years in 2019, corresponding to nearly 750,000 deaths globally [1]. The burden is disproportionately concentrated in South Asia and sub-Saharan Africa, where delayed access to care, limited healthcare resources, malnutrition, incomplete immunization coverage, and socioeconomic inequalities continue to contribute to poor outcomes [1]. The uploaded manuscript also frames CAP as a major cause of pediatric morbidity and mortality, especially in low-resource settings.

Childhood CAP encompasses a wide clinical spectrum, ranging from mild, self-limiting lower respiratory tract infection to severe disease complicated by respiratory failure, sepsis, pleural involvement, or the need for intensive care. Its etiology is heterogeneous and influenced by age, geographic setting, seasonality, vaccination coverage, host immune status, and comorbid conditions [1,3]. Viral pathogens are particularly common in infants and preschool-aged children, whereas bacterial pathogens become increasingly relevant in older children and in severe or complicated disease [1,3]. Among bacterial causes, *Streptococcus pneumoniae* remains a major pathogen despite widespread pneumococcal conjugate vaccination, while *Haemophilus influenzae* type b continues to contribute to disease burden in regions with suboptimal vaccine coverage [1,3]. Atypical pathogens, especially *Mycoplasma pneumoniae*, are more frequently identified in school-aged children and adolescents, whereas opportunistic organisms such as *Pneumocystis jirovecii* remain important causes of severe pneumonia in immunocompromised children [1,3].

Although pathogen eradication remains central to CAP management, increasing attention has focused on the role of the host inflammatory response in determining disease severity and clinical outcomes [3,4]. CAP pathogenesis reflects a dynamic interaction between invading microorganisms and the host immune system. Following infection of the lower respiratory tract, activation of innate immune pathways promotes the release of cytokines, chemokines, and other inflammatory mediators that recruit immune cells and support pathogen clearance [4,5]. However, when this response becomes excessive, prolonged, or dysregulated, inflammation may contribute to alveolar-capillary barrier disruption, increased vascular permeability, pulmonary edema, impaired gas exchange, and local or systemic complications [4,5]. Thus, in selected patients, disease progression may be driven not only by microbial replication but also by immune-mediated tissue injury.

This recognition has stimulated interest in adjunctive therapeutic strategies aimed at modulating the host response in addition to targeting the causative pathogen [4,5,6]. Corticosteroids are among the most extensively studied immunomodulatory agents in infectious and inflammatory pulmonary diseases. Through genomic and non-genomic mechanisms, they suppress pro-inflammatory cytokine production, reduce leukocyte recruitment and activation, stabilize endothelial and epithelial barriers, and attenuate tissue inflammation [4,6,7]. These effects provide a biologically plausible rationale for their use as adjunctive therapy in pneumonia, particularly in severe disease or in clinical phenotypes characterized by exaggerated inflammation.

Over the past two decades, corticosteroids have been increasingly investigated in respiratory infections and other inflammatory pulmonary conditions, where they may reduce inflammation-mediated tissue injury and accelerate clinical recovery [6]. In adult patients with CAP, several studies and meta-analyses suggest that adjunctive corticosteroid therapy may improve selected outcomes, including faster clinical stabilization and reduced inflammatory complications, especially in severe disease [8,9]. However, extrapolating these findings to children is problematic. Pediatric patients differ from adults in immune maturation, pathogen distribution, clinical presentation, comorbidity profile, and treatment response [3]. Moreover, the etiologic spectrum of childhood CAP is distinct, with a greater contribution of viral and atypical pathogens, potentially influencing both the inflammatory phenotype and the expected benefit of immunomodulatory therapy.

Consequently, the role of systemic corticosteroids in pediatric CAP remains incompletely defined and clinically controversial [4,10]. Although corticosteroids are widely used in several pediatric respiratory conditions, particularly asthma and wheezing disorders, their routine use in childhood CAP is not currently supported by definitive evidence. Key uncertainties persist regarding which patients are most likely to benefit; the optimal timing of initiation; the most appropriate drug, dose, and duration; and the balance between potential clinical benefit and treatment-related harm [10]. These uncertainties have contributed to variability in clinical practice and to the absence of clear, pediatric-specific recommendations for corticosteroid use in CAP.

An important distinction is required between pediatric CAP, in which the role of systemic corticosteroids remains uncertain, and clinical conditions in which corticosteroids are prescribed for a separate, established indication. Children with CAP and concomitant wheezing or asthma exacerbation represent a distinct subgroup because any benefit of corticosteroids is likely to result primarily from improvement in bronchospasm and airway inflammation rather than from a direct effect on the pneumonic process [10]. Similarly, *P. jirovecii* pneumonia (PJP) and severe or critical COVID-19 are pathogen-specific conditions with separate therapeutic frameworks. Adjunctive corticosteroids are established in moderate-to-severe HIV-associated PJP with clinically significant hypoxemia and are recommended in severe or critical COVID-19 requiring oxygen or ventilatory support [3]. These situations should therefore not be interpreted as evidence supporting routine corticosteroid use in pediatric CAP more broadly.

Accordingly, the principal focus of the present review is pediatric CAP without an independent, guideline-supported indication for systemic corticosteroids. The evaluation of clinical efficacy, including fever resolution, length of stay, respiratory improvement, treatment failure, readmission, and safety, primarily concerns non-wheezing CAP, severe or refractory bacterial or atypical pneumonia, complicated pneumonia, and other settings in which the role of corticosteroids remains uncertain. Wheezing-associated pneumonia, COVID-19, and PJP are discussed separately because their mechanisms, indications, treatment thresholds, and supporting evidence differ substantially from those of general pediatric CAP.

This distinction is also important when interpreting observational studies. Cohorts that combine children with bacterial CAP, viral wheezing, asthma exacerbation, COVID-19, PJP, or other immunocompromising conditions may produce misleading estimates of corticosteroid efficacy. An apparent benefit may be driven by treatment of airway obstruction or by inclusion of conditions for which corticosteroids are already indicated, whereas neutral or harmful associations may reflect unmeasured differences in severity, immune status, pathogen burden, or timing of treatment. Studies should therefore report these subgroups separately and adjust, where possible, for wheezing status, bronchodilator use, asthma history, immune status, microbiological diagnosis, oxygen requirement, and disease severity.

Within this framework, wheezing-associated pneumonia, severe or critical COVID-19, and PJP should be regarded as special therapeutic scenarios rather than as representative phenotypes of pediatric CAP. Their separate presentation earlier in the manuscript clarifies that the central unresolved question is whether systemic corticosteroids provide benefit in children with CAP who do not have one of these established or independently steroid-responsive indications.

Given these unresolved issues, a comprehensive appraisal of the available evidence is warranted. This narrative review critically examines the use of systemic corticosteroids in children with CAP, focusing on the biological rationale, clinical efficacy, safety profile, therapeutic regimens, timing of administration, and potential role in specific pediatric phenotypes, including severe CAP, *M. pneumoniae* pneumonia (MPP), complicated pneumonia, viral pneumonia, wheezing-associated presentations, and immunocompromised hosts. By synthesizing the current evidence, this review aims to clarify areas of clinical uncertainty, identify knowledge gaps, and highlight priorities for future research toward more precise, phenotype-driven, and evidence-based therapeutic strategies for pediatric pneumonia. The present review was conceived to complement, rather than replicate, the earlier Cochrane review and the 2026 pediatric systematic review and meta-analysis [3,10]. The Cochrane review evaluated corticosteroids for pneumonia across heterogeneous populations and was primarily designed to assess treatment efficacy using predefined outcomes. The more recent 2026 systematic review provides an updated quantitative synthesis of pediatric studies, but its scope is necessarily constrained by the eligibility criteria and outcomes suitable for meta-analysis. In contrast, the present narrative review addresses several clinically relevant questions that cannot be adequately examined through pooled estimates alone. Specifically, this review integrates evidence across distinct pediatric pneumonia phenotypes, including severe and refractory MPP, wheezing-associated presentations, viral pneumonia, pleural complications, ARDS, and pneumonia in immunocompromised children. It also examines how pathogen biology, host inflammatory responses, diagnostic uncertainty, timing within the disease course, corticosteroid regimen, biomarker interpretation, and confounding by indication may modify apparent treatment effects. Particular attention is given to the limitations of proposed biomarker thresholds, the distinction between prognostic markers and predictors of corticosteroid responsiveness, and the difficulty of extrapolating adult findings to children. The added value of this review therefore lies in its mechanistic and phenotype-oriented interpretation of the evidence; its comparison of positive, neutral, and potentially harmful findings; and its emphasis on why apparently conflicting results may arise across studies. Rather than providing another pooled estimate of efficacy, the review aims to clarify where evidence is strongest, where it remains inconsistent or absent, and which clinical and biological variables should be incorporated into future pediatric trials. This broader synthesis is intended to help clinicians interpret the conclusions of previous systematic reviews in the context of individual patient characteristics and current limitations in diagnosis, risk stratification, and treatment standardization.

## 2. Methods

### 2.1. Review Design and Scope

This narrative review was designed to summarize and critically appraise the available evidence on systemic corticosteroids as adjunctive therapy in pediatric CAP. Particular attention was given to severe bacterial CAP, atypical CAP—especially MPP—and selected clinical phenotypes in which an excessive or dysregulated host inflammatory response may contribute substantially to disease severity. These phenotypes included viral CAP, parapneumonic effusion, empyema, wheezing-associated CAP, and pneumonia in immunocompromised children.

Although this work was conducted as a narrative rather than a systematic review, a structured and reproducible approach was used for literature identification, study selection, data extraction, and qualitative synthesis.

### 2.2. Information Sources and Search Period

A structured literature search was conducted in PubMed/MEDLINE, Scopus, and Web of Science. Publications indexed or available up to 30 June 2026 were considered.

The electronic search was supplemented by manually screening the reference lists of eligible primary studies, relevant systematic reviews, meta-analyses, clinical reviews, and other key articles. Citation tracking was also performed for selected influential publications when relevant. The search was intended primarily to identify pediatric evidence. Adult studies were considered only when they provided relevant mechanistic, pharmacological, safety, or clinical context that could not be adequately obtained from pediatric studies.

No restrictions were applied regarding geographical setting. Publications in English only were eligible.

### 2.3. Search Strategy

The search strategy combined controlled vocabulary terms, where available, with free-text terms relating to pediatric pneumonia and systemic corticosteroid therapy. Search concepts included: (1) the pediatric population; (2) pneumonia and its relevant clinical phenotypes or etiologies; and (3) systemic corticosteroids or glucocorticoid therapy.

The following terms and their variants were used alone or in combination: “pediatric pneumonia”, “child”, “children”, “adolescent”, “community-acquired pneumonia”, “severe pneumonia”, “corticosteroids”, “systemic corticosteroids”, “glucocorticoids”, “methylprednisolone”, “dexamethasone”, “prednisone”, “prednisolone”, “adjunctive therapy”, “immunomodulation”, “*Mycoplasma pneumoniae*”, “*Mycoplasma pneumoniae* pneumonia”, “viral pneumonia”, “parapneumonic effusion”, and “empyema”.

Boolean operators, truncation, phrase searching, and database-specific subject headings were used as appropriate. A representative PubMed search strategy was:
((“Pneumonia”[Mesh] OR pneumonia*[Title/Abstract] OR “community-acquired pneumonia”[Title/Abstract] OR “severe pneumonia”[Title/Abstract] OR “*Mycoplasma pneumoniae* pneumonia”[Title/Abstract] OR “viral pneumonia”[Title/Abstract] OR “parapneumonic effusion”[Title/Abstract] OR empyema[Title/Abstract]) AND (“Adrenal Cortex Hormones”[Mesh] OR corticosteroid*[Title/Abstract] OR glucocorticoid*[Title/Abstract] OR methylprednisolone[Title/Abstract] OR dexamethasone[Title/Abstract] OR prednisone[Title/Abstract] OR prednisolone[Title/Abstract]) AND (“Child”[Mesh] OR “Infant”[Mesh] OR “Adolescent”[Mesh] OR child*[Title/Abstract] OR pediatric*[Title/Abstract] OR paediatric*[Title/Abstract] OR adolescent*[Title/Abstract])).

The syntax was adapted for Scopus and Web of Science.

### 2.4. Eligibility Criteria

Primary studies were eligible when they evaluated systemic corticosteroid therapy in children or adolescents aged 0–18 years with clinically diagnosed, radiologically diagnosed, or microbiologically confirmed pneumonia.

Eligible study designs included randomized controlled trials, non-randomized clinical trials, prospective or retrospective cohort studies, case–control studies, and other observational comparative analyses. Systematic reviews and meta-analyses were included to identify relevant primary studies and to provide an overview of the broader evidence base. Case reports and small case series were considered only when they addressed uncommon clinical phenotypes, unusual complications, or treatment regimens for which higher-level pediatric evidence was unavailable.

Studies were considered eligible when they reported at least one clinically relevant outcome, including:

1. Duration of fever or time to defervescence;
2. Respiratory symptoms or clinical stabilization;
3. Oxygen requirement or need for respiratory support;
4. Admission to intensive care;
5. Length of hospital stay;
6. Treatment failure or escalation of therapy;
7. Radiological progression or resolution;
8. Changes in inflammatory biomarkers;
9. Pleural or pulmonary complications;
10. Mortality;
11. Readmission; or
12. Corticosteroid-related adverse events.

Studies were excluded when they:

1. Included exclusively adult populations and did not provide relevant mechanistic or contextual information applicable to pediatric practice;
2. Evaluated non-infectious pulmonary diseases;
3. Assessed inhaled corticosteroids without systemic corticosteroid administration;
4. Did not separately report pediatric data when pediatric and adult participants were combined;
5. Did not evaluate corticosteroid exposure in relation to pneumonia-related clinical, laboratory, radiological, or safety outcomes;
6. Were editorials, commentaries, letters without original data, or narrative opinion articles that did not contribute relevant contextual information; or
7. Were duplicate publications or secondary reports of an already included study without additional relevant data.

Selected mechanistic studies that did not directly evaluate corticosteroid treatment were retained separately when they provided relevant information on pneumonia immunopathogenesis, host inflammatory responses, or corticosteroid pharmacology. These studies were used for biological context and were not treated as clinical effectiveness studies.

### 2.5. Study Selection and Screening

All records retrieved from the electronic databases were exported to Rayyan (Rayyan Systems Inc., Cambridge, MA, USA; web version),. Duplicate records were identified electronically and subsequently verified manually before screening.

Study selection was conducted in two stages. First, the titles and abstracts of all unique records were screened against the predefined eligibility criteria. Records that were clearly unrelated to pediatric pneumonia, systemic corticosteroid therapy, or the outcomes of interest were excluded at this stage.

Second, the full texts of potentially eligible articles were retrieved and assessed. Full-text articles were excluded when they did not meet the population, intervention, study-design, or outcome criteria. The principal reason for exclusion was recorded for each article excluded after full-text assessment.

Screening was performed independently by SR and ER. Disagreements regarding eligibility were resolved through discussion and consensus. When consensus could not be reached, a third reviewer (GZ) made the final decision.

The study-selection process resulted in approximately 286 records identified through database searching and 18 additional records identified through reference-list screening and citation tracking. After removal of 74 duplicate records, 230 titles and abstracts were screened. Of these, 81 full-text articles were assessed for eligibility, and 64 publications were included in the final narrative synthesis.

### 2.6. Data Extraction

A standardized data-extraction form was developed before evidence synthesis. For each eligible clinical study, the following information was extracted:

1. Author and year of publication;
2. Country and clinical setting;
3. Study design;
4. Sample size;
5. Patient age and relevant comorbidities;
6. Diagnostic criteria for pneumonia;
7. Pneumonia phenotype and suspected or confirmed etiology;
8. Criteria used to define severe, refractory, or complicated disease;
9. Corticosteroid agent;
10. Route of administration;
11. Dose and dosing frequency;
12. Treatment duration and tapering strategy;
13. Timing of corticosteroid initiation;
14. Comparator or standard-care treatment;
15. Concomitant antimicrobial and supportive therapies;
16. Clinical, laboratory, radiological, and safety outcomes;
17. Duration of follow-up; and
18. Principal findings and limitations.

Data extraction was performed by SDT and checked by MF. Discrepancies were resolved by consensus. When relevant information was incomplete or unclear, the study was retained only if sufficient data were available to interpret its population, corticosteroid exposure, and reported outcomes.

For systematic reviews and meta-analyses, information was extracted on the review population, eligibility criteria, number and type of included studies, corticosteroid interventions, principal outcomes, and major conclusions. Care was taken to avoid counting the same patient populations more than once when primary studies were also discussed individually.

### 2.7. Evidence Appraisal and Synthesis

Because this was a narrative review, no prospective protocol was registered, and formal meta-analytic pooling was not undertaken. A standardized numerical quality score was also not applied. Nevertheless, the strength and applicability of individual studies were considered qualitatively according to study design, sample size, patient-selection methods, comparability of treatment groups, adjustment for confounding, definition of pneumonia and disease severity, microbiological confirmation, completeness of outcome reporting, and risk of confounding by indication.

Randomized studies and systematic reviews were given greater interpretive weight than uncontrolled or retrospective observational studies. Findings from adult studies were clearly distinguished from pediatric evidence and were used only for mechanistic or contextual interpretation when pediatric data were limited.

Studies were grouped according to the following thematic domains:

1. Biological rationale and immunopathogenesis;
2. Clinical indications and potentially responsive phenotypes;
3. Biomarkers and risk stratification;
4. Corticosteroid agent, dose, route, timing, and duration;
5. Clinical efficacy and patient outcomes;
6. Safety and adverse effects; and
7. Evidence limitations and priorities for future research.

Within each domain, findings were compared according to study design, patient population, pneumonia etiology, severity, corticosteroid regimen, timing of administration, comparator treatment, and outcome definitions. Particular attention was given to clinically meaningful endpoints, including time to defervescence, oxygen or respiratory-support requirements, length of hospital stay, treatment failure, radiological progression, inflammatory-marker trends, development of complications, mortality, readmission, and treatment-related adverse events.

Because substantial clinical and methodological heterogeneity was present across the included studies, results were synthesized descriptively rather than quantitatively. The synthesis aimed to identify consistent findings, areas of disagreement, potential sources of heterogeneity, and evidence gaps relevant to a more precise, phenotype-based approach to systemic corticosteroid therapy in pediatric pneumonia.

## 3. Corticosteroids in Pediatric Pneumonia

Systemic corticosteroids have been increasingly investigated as adjunctive therapy in pediatric pneumonia because, in selected children, disease severity may be driven not only by pathogen burden but also by an excessive or dysregulated host inflammatory response [3,4,11,12,13,14]. This concept is particularly relevant in severe CAP, refractory MPP, complicated CAP with pleural involvement, and selected phenotypes characterized by marked systemic inflammation. However, the potential benefits of corticosteroids must be balanced against important uncertainties regarding patient selection, timing, dosage, and safety. Therefore, their use should be considered within a phenotype-based framework rather than as routine therapy for all children with pneumonia.

### 3.1. Biological Rationale and Mechanisms of Action

The rationale for adjunctive corticosteroid therapy in pediatric CAP is based on the recognition that pulmonary injury and clinical deterioration may result from both pathogen-related damage and the host inflammatory response [3,4,11,12,13,14]. Inflammation is essential for pathogen containment and clearance; however, when it becomes excessive, prolonged, or poorly regulated, it may contribute to epithelial and endothelial injury, disruption of the alveolar-capillary barrier, increased vascular permeability, pulmonary edema, impaired ventilation–perfusion matching, and progressive respiratory failure [15,16,17,18].

Following infection of the lower respiratory tract, pathogen-associated molecular patterns and damage-associated molecular patterns activate immune pathways within the airway and alveolar compartments. This activation promotes recruitment of neutrophils, macrophages, monocytes, lymphocytes, and other immune cells and induces the production of interleukin-6, tumor necrosis factor-α, interferons, chemokines, and additional inflammatory mediators [15,16,17,18]. These responses initially support host defense, but persistent activation may amplify tissue injury beyond that caused by the pathogen itself.

Pneumonia pathogenesis is therefore unlikely to result from a single mechanism. Viral cytopathic effects, bacterial toxins, enzymes, membrane components, and other virulence-associated factors may directly injure epithelial or endothelial cells or initiate inflammatory cascades [5,16,19]. At the same time, infected, stressed, or dying host cells release nucleic acids, intracellular proteins, peptides, oxidized lipids, extracellular vesicles, cytokines, chemokines, and other danger signals that may propagate inflammation beyond the initially infected cells [5,7,16,17,18]. In severe disease, these host-derived mediators may continue to sustain pulmonary inflammation even after the burden of viable microorganisms has declined.

This interaction is particularly relevant in influenza, COVID-19, and severe or refractory MPP, in which extensive tissue injury may reflect both direct pathogen-related effects and exaggerated immune activation [14,19,20]. In MPP, attachment to respiratory epithelial cells, disruption of ciliary function, oxidative stress, membrane injury, and release of intracellular danger signals may combine with sustained immune activation to produce severe pulmonary and extrapulmonary manifestations [14,16,19,20]. Thus, pathogen detection alone does not fully define the dominant mechanism of injury at a given stage of disease.

The conventional distinction between innate and adaptive immunity remains useful, but it does not fully describe the overlapping cellular networks involved in disease progression and recovery. Neutrophils, macrophages, monocytes, dendritic cells, natural killer cells, T and B lymphocytes, epithelial cells, endothelial cells, and stromal cells interact through cytokines, chemokines, receptors, and metabolic signals [7,15,16,17,18]. These networks participate not only in pathogen control but also in removal of damaged material, limitation of collateral injury, resolution of inflammation, and tissue repair. Severe disease should therefore be viewed as a dynamic network process rather than as a simple sequential transition from innate to adaptive immunity.

The protein-homeostasis-system hypothesis offers a broader conceptual interpretation in which the magnitude and characteristics of the immune response are related to the amount and biochemical properties of disease-inducing substances. Within this framework, relevant stimuli may include microbial nucleic acids, proteins, peptides, toxins, enzymes, cell-wall or membrane components, extracellular vesicles, and endogenous products released from damaged host cells. This model may help integrate pathogen burden, tissue injury, and immune activation; however, it remains hypothetical and should not be considered an established replacement for conventional immunopathological models. In particular, the proposal that pathogenic peptides released from infected cells are the principal disease-inducing substances in viral or *M. pneumoniae* pneumonia remains insufficiently validated. Direct evidence defining their identity, concentrations, tissue distribution, receptor interactions, and causal role is limited. Similarly, cytokine storm is unlikely to result from a single immune-cell population, because severe hyperinflammation generally involves multiple immune and non-immune cell types and varies according to pathogen and host characteristics [16,17,18].

Different respiratory pathogens may nevertheless produce overlapping pathological and clinical manifestations. Coronaviruses, adenoviruses, parainfluenza viruses, *M. pneumoniae*, *S. pneumoniae*, and *H. influenzae* differ in cellular tropism, replication strategy, virulence factors, and interactions with host immunity [16,17,18]. However, each may ultimately produce epithelial and endothelial injury, leukocyte recruitment, alveolar edema, increased permeability, impaired gas exchange, and, in severe cases, acute respiratory distress syndrome. Similar downstream pathways may also be activated by non-infectious insults, including aspiration, blunt chest trauma, severe burns, toxic inhalation, pancreatitis, amniotic fluid embolism, multiple injuries, and autoimmune disease. These conditions may release endogenous danger signals and activate inflammatory pathways that converge on diffuse alveolar and endothelial injury.

From this perspective, disease severity may depend on the magnitude and persistence of the injurious stimulus, its anatomical distribution, the effectiveness of pathogen control, the susceptibility of pulmonary cells, and the host’s ability to limit both infection and collateral immune-mediated damage. Mild disease may remain self-limited when pathogen- and host-derived stimuli are rapidly controlled. Severe pneumonia or ARDS may develop when microbial burden is greater, clearance is delayed, tissue injury is extensive, or inflammatory amplification exceeds the host’s regulatory capacity.

The respiratory tract should also be considered an ecological system rather than a sterile compartment. Microbial communities are present throughout the upper respiratory tract and, at lower biomass, in the lower airways. Their composition varies with age, environmental exposures, diet, household conditions, hygiene practices, antibiotic use, vaccination, and underlying health status [21,22]. Bacteria, viruses, and fungi may coexist with the host without causing clinical disease.

Accordingly, detection of a microorganism in a respiratory specimen does not necessarily establish causation. *S. pneumoniae* and *H. influenzae* commonly colonize the upper respiratory tract in children, while *M. pneumoniae* DNA may be detected in asymptomatic individuals or persist after clinical recovery [23]. Respiratory viruses may also be detected during asymptomatic infection or prolonged post-infectious shedding [24]. Positive polymerase chain reaction, culture, antigen, or serological results should therefore be interpreted in relation to specimen type, microbial load, timing of sampling, clinical presentation, radiological findings, host inflammatory responses, and the presence of alternative or co-detected pathogens.

It is important to distinguish microbiota membership, colonization, infection, and disease. Colonization refers to the presence or replication of a microorganism without clinically significant tissue injury. Infection involves interaction with host tissues and activation of a host response, but it may remain asymptomatic or subclinical. Disease develops when this interaction produces sufficient cellular injury, inflammation, or physiological dysfunction to cause clinical manifestations [21,22,23,24]. The transition from carriage to disease may be influenced by microbial density, virulence factors, disruption of the epithelial barrier, viral–bacterial interactions, antibiotic exposure, changes in microbial competition, or impaired host defenses [5,16,21,22].

Respiratory infections caused by human-adapted pathogens may occur as seasonal epidemics, periodic outbreaks, or pandemics. During outbreaks of influenza, COVID-19, or *M. pneumoniae* infection, most children experience asymptomatic, mild, or self-limited illness, whereas a smaller proportion develop pneumonia, and only a minority progress to hypoxemia, respiratory failure, or ARDS [1,3,19,23,24,25]. Children may contribute substantially to transmission because of close social contacts, limited pre-existing immunity, and asymptomatic or mildly symptomatic shedding. For acute viral infections, however, the terms asymptomatic or presymptomatic infection and viral shedding are generally more precise than stable colonization or reservoir status.

These epidemiological and diagnostic considerations are important when interpreting studies of corticosteroid therapy. Hospital-based cohorts represent a selected subgroup at the severe end of the disease spectrum and may not reflect the much larger population with mild or asymptomatic infection. Misclassification of colonization, prolonged detection, or incidental coinfection as the cause of pneumonia may obscure pathogen-specific treatment effects. Observed associations may also be influenced by epidemic phase, circulating strain, age distribution, prior immunity, vaccination status, symptom duration, referral patterns, and criteria for hospitalization.

Corticosteroids may attenuate inflammation through genomic and non-genomic mechanisms. By binding to intracellular glucocorticoid receptors, they regulate transcription of inflammatory genes, suppress production of pro-inflammatory cytokines, inhibit leukocyte activation and migration, and reduce epithelial and endothelial permeability [4,6,7]. Their more rapid non-genomic effects may also influence vascular tone, intracellular signaling, and inflammatory-cell function. Corticosteroids additionally suppress T-cell activation, proliferation, and cytokine production, which may be relevant in severe or refractory MPP and other conditions characterized by prominent immune-mediated injury [14,19,20,21].

The potential benefit of corticosteroids is therefore likely to depend on the dominant mechanism and temporal phase of injury. They may be most useful when dysregulated host inflammation has become a major driver of deterioration despite adequate pathogen-directed treatment. Conversely, they may be ineffective or harmful when active microbial replication remains insufficiently controlled and immune responses are still required for clearance [10,14]. At present, no validated biomarker or clinical test can reliably identify the transition from predominantly pathogen-driven disease to predominantly host-mediated injury.

Future studies should therefore integrate quantitative pathogen assessment with longitudinal characterization of pathogen- and host-derived mediators, immune-cell activation, cytokine networks, respiratory microbiota, and markers of epithelial and endothelial injury. Such approaches may clarify why most respiratory infections remain self-limited, whereas a minority progress to severe pneumonia or ARDS, and may identify biologically defined subgroups in which immunomodulatory therapy is most likely to be beneficial.

The use of corticosteroids in infectious pneumonia requires careful balance between preservation of antimicrobial host defenses and limitation of inflammation-mediated lung injury. Concern regarding corticosteroid therapy is justified when direct pathogen-induced cytopathy, uncontrolled microbial replication, or impaired pathogen clearance remains the predominant mechanism of disease. However, in severe pneumonia and pneumonia-associated ARDS, clinical deterioration may also be driven by an excessive or dysregulated host response, with alveolar–capillary barrier injury, pulmonary edema, and impaired gas exchange. In these circumstances, appropriately timed immunomodulation may be beneficial, provided that effective pathogen-directed therapy and adequate supportive care are maintained. The decision to initiate corticosteroids should therefore remain the responsibility of the treating physician and should be individualized according to disease severity, clinical trajectory, suspected etiology, inflammatory phenotype, oxygen or ventilatory requirements, comorbidities, and the potential risks of immunosuppression. Published guidelines and reviews, including the present article, should be interpreted as decision-support tools rather than prescriptive substitutes for clinical judgment. At present, the absence of validated biomarkers identifying the transition from predominantly pathogen-driven injury to predominantly host-mediated inflammation further reinforces the need for multidisciplinary assessment and repeated clinical reassessment when determining the timing, dose, and duration of corticosteroid therapy.

### 3.2. Clinical Indications for Corticosteroid Therapy

Current evidence does not support the routine use of systemic corticosteroids in all children with CAP [10,26,27,28]. Instead, available data suggest that corticosteroid therapy may be most appropriate in selected clinical scenarios characterized by severe inflammation, respiratory compromise, progressive disease, or inadequate response to standard antimicrobial treatment. The decision to use corticosteroids should therefore be individualized and based on disease phenotype, clinical trajectory, inflammatory burden, and potential risks.

The most consistent pediatric evidence relates to severe or refractory MPP, including macrolide-resistant forms [11,14,29]. In these patients, disease progression is thought to result from both microbial factors and an exaggerated host immune response. Corticosteroids have been used in children with persistent fever, worsening respiratory symptoms, progressive radiographic abnormalities, hypoxemia, or clinical deterioration despite appropriate antimicrobial therapy [13,30]. In this context, corticosteroids are generally considered as adjunctive or rescue therapy rather than first-line treatment for uncomplicated disease.

Beyond MPP, corticosteroids have been explored in severe CAP associated with marked respiratory distress, extensive pulmonary involvement, systemic inflammation, or evolving respiratory failure [6,31]. In these situations, attenuation of excessive inflammation may contribute to faster clinical stabilization and may reduce progression of lung injury. However, evidence remains heterogeneous, and the benefit appears most plausible in children with severe inflammatory phenotypes rather than in unselected hospitalized patients.

Complicated CAP represents another potential indication. In children with parapneumonic pleural effusion or empyema, corticosteroids—particularly dexamethasone—have been investigated as adjunctive anti-inflammatory therapy [6,31]. By limiting pleural and pulmonary inflammation, corticosteroids may shorten fever duration, reduce inflammatory burden, and potentially decrease length of hospital stay or need for invasive procedures. Nevertheless, their role in this setting remains adjunctive and should be considered alongside appropriate antimicrobial therapy, drainage when indicated, and careful clinical monitoring.

Children with CAP and concomitant wheezing or asthma exacerbation represent a distinct subgroup in which systemic corticosteroids are frequently prescribed [12]. In such cases, any observed benefit may reflect improvement in airway inflammation and bronchospasm rather than a direct effect on pneumonia itself. This distinction is important because misclassification between viral wheezing illness, asthma exacerbation, and bacterial CAP may partly explain conflicting findings across pediatric studies.

Conversely, routine corticosteroid administration in uncomplicated CAP is not currently supported. In children without severe disease, hypoxemia, wheezing, refractory MPP, complicated CAP, or evidence of marked inflammation, the likelihood of benefit appears limited, while potential adverse effects remain relevant [10,27]. Therefore, corticosteroids should not be regarded as standard adjunctive therapy for all pediatric CAP but rather as a targeted intervention for carefully selected patients.

### 3.3. Biomarkers for Patient Selection and Risk Stratification

Appropriate patient selection remains one of the major challenges in corticosteroid therapy for pediatric CAP. Because corticosteroids are unlikely to provide uniform benefit across all children with CAP, considerable interest has focused on identifying biomarkers that may help distinguish children with self-limited infection from those with severe, refractory, or hyperinflammatory disease. Ideally, biomarkers could support clinical decision-making by identifying patients at higher risk of progression and those most likely to benefit from immunomodulatory treatment.

Several inflammatory and tissue injury markers have been associated with severe or refractory CAP, particularly in MPP. These include C-reactive protein (CRP), lactate dehydrogenase (LDH), ferritin, transaminases, and cytokines such as IL-6 and IL-18 [32,33,34]. Elevated levels of these markers may reflect systemic inflammation, immune activation, tissue injury, or macrophage activation, and have been correlated in some studies with persistent fever, extensive radiological involvement, hypoxemia, and delayed clinical recovery [32,33,34,35].

Among currently available biomarkers, LDH has emerged as one of the most promising indicators of pulmonary tissue injury. Increased LDH levels have been associated with extensive radiographic abnormalities, persistent fever, severe inflammatory responses, and refractory MPP, suggesting a potential role in identifying children who may require closer monitoring or more aggressive immunomodulatory therapy [11,14,35]. CRP may also support assessment of systemic inflammatory burden, although it lacks specificity and should not be interpreted in isolation. Ferritin may be useful in identifying hyperinflammatory phenotypes, particularly when systemic inflammation appears disproportionate to the clinical course [14].

Several studies have proposed threshold values to assist risk stratification and guide consideration of corticosteroid therapy. Suggested cutoffs include LDH ≥ 590 IU/L, CRP ≥ 44.45 mg/L, and ferritin ≥ 411 ng/L, particularly in children with severe or refractory MPP being considered for high-dose corticosteroid treatment [14]. However, these thresholds have not been consistently validated across different populations, age groups, healthcare settings, or CAP etiologies. They should therefore be regarded as supportive indicators rather than definitive criteria for initiating corticosteroids.

Cytokine markers, including IL-6 and IL-18, may provide additional information regarding the intensity of immune activation and inflammatory injury [33,34]. However, their clinical utility remains limited by restricted availability, lack of standardized cutoffs, variability in assay methods, and insufficient validation in routine pediatric practice. At present, no single biomarker has sufficient accuracy to independently determine whether corticosteroids should be administered.

Therefore, biomarker interpretation should be integrated with the overall clinical picture, including disease severity, oxygen requirement, radiological progression, microbiological diagnosis, persistence of fever, and response to antimicrobial therapy [11,14]. Serial measurement may be particularly useful for monitoring disease evolution and treatment response, especially in children with severe or refractory disease.

The clinical role of inflammatory biomarkers in pediatric CAP should be interpreted according to whether they are being used for prediction, prognosis, or treatment monitoring. At present, CRP, LDH, ferritin, IL-6, and IL-18 are primarily prognostic or supportive markers rather than validated predictive biomarkers of corticosteroid responsiveness. CRP reflects systemic inflammatory burden and may help identify children with more severe disease, but it is nonspecific and cannot reliably distinguish bacterial, viral, or atypical CAP or determine whether corticosteroid therapy will be beneficial. LDH is more closely associated with pulmonary tissue injury and has been repeatedly linked to severe or refractory MPP, extensive radiological involvement, and delayed clinical recovery [14]. It may therefore have prognostic value, although its ability to predict a therapeutic response to corticosteroids has not been prospectively established.

Ferritin may indicate a hyperinflammatory phenotype and could support recognition of children at risk of severe systemic inflammation [14]. However, elevated ferritin is not specific to CAP and may also occur in other infectious, inflammatory, hepatic, or macrophage-activation conditions. IL-6 and IL-18 are biologically relevant markers of immune activation and have been associated with disease severity in selected studies, but their routine clinical use is limited by restricted assay availability, differences in laboratory methods, biological variability, and the absence of standardized pediatric reference ranges [33,34].

Serial measurements of CRP, LDH, ferritin, or cytokines may be useful for treatment monitoring when interpreted together with fever resolution, oxygen requirement, respiratory status, and radiological progression. A decline after therapy may support clinical improvement, but corticosteroids directly suppress inflammatory-marker concentrations; therefore, biomarker reduction alone should not be considered proof of microbiological resolution or treatment success.

Several proposed thresholds, including LDH values around 590 IU/L, CRP values around 44 mg/L, and ferritin values around 411 ng/mL, have been reported in selected populations, particularly children with severe or refractory MPP [14]. These cut-offs were generally derived retrospectively from single-center cohorts and have not been consistently validated across age groups, geographic settings, etiologies, assay platforms, or definitions of disease severity. Consequently, no biomarker or cut-off currently has sufficient sensitivity, specificity, or external validation to independently guide corticosteroid initiation, dose selection, or treatment duration. Biomarkers should therefore be regarded as adjuncts to, rather than replacements for, comprehensive clinical assessment. Prospective multicenter studies are needed to determine whether single markers or combined biomarker panels can reliably predict corticosteroid responsiveness and improve phenotype-based treatment selection.

Table 1 summarizes the principal biomarkers investigated for patient selection and risk stratification in pediatric CAP, emphasizing their biological relevance, clinical associations, proposed thresholds, and potential practical utility.

### 3.4. Therapeutic Regimens, Timing, and Duration of Therapy

Considerable variability exists in the corticosteroid regimens used for pediatric pneumonia, reflecting differences in CAP etiology, disease severity, inflammatory burden, clinical setting, and local practice patterns. At present, no universally accepted pediatric protocol has been established, and most available treatment approaches are derived from observational studies, small clinical trials, and extrapolation from specific disease phenotypes rather than from large, standardized randomized controlled trials [3,36]. Accordingly, corticosteroid therapy should be individualized, with careful consideration of the suspected pathogen, severity of illness, timing of disease progression, and potential risk of adverse effects.

Methylprednisolone is among the most frequently used systemic corticosteroids in hospitalized children with severe or refractory CAP. It is generally administered intravenously, with reported doses ranging from 2 to 30 mg/kg/day [11,30]. Lower-dose regimens are more commonly used in children with moderate inflammatory activity, whereas higher-dose approaches have been described in severe or refractory disease characterized by persistent fever, extensive radiological involvement, hypoxemia, or markedly elevated inflammatory biomarkers [11,14,30]. In particular, pulse methylprednisolone regimens, usually 10–30 mg/kg/day, have been associated with rapid defervescence and clinical improvement in children with refractory MPP and other hyperinflammatory presentations [13,14,37,38].

Oral corticosteroids, including prednisone and prednisolone, are more often used in milder disease, during step-down therapy after initial intravenous treatment, or in children with overlapping wheezing or asthma exacerbation phenotypes. Weight-based regimens have been reported, although dosing schedules, duration of therapy, and tapering strategies vary substantially across studies and institutions [11,30,39]. This variability limits direct comparison between studies and contributes to uncertainty regarding the optimal oral regimen.

Dexamethasone has been mainly evaluated in children with parapneumonic pleural effusion and empyema. Because of its potent anti-inflammatory activity and prolonged biological half-life, dexamethasone may be particularly useful in inflammatory pleural disease, where adjunctive treatment has been associated in some studies with faster clinical improvement and shorter hospitalization [6,31]. However, its use should be considered as part of a comprehensive management strategy that includes appropriate antimicrobial therapy and drainage procedures when clinically indicated.

The optimal timing of corticosteroid initiation remains uncertain. Some studies suggest that early administration, particularly within the first 24–36 h of hospitalization, may be associated with faster fever resolution and earlier clinical improvement [11,25,40]. However, other data indicate that corticosteroids may be most effective when introduced after it becomes clear that immune-mediated inflammation is contributing to disease progression, such as in children with persistent fever, worsening radiographic abnormalities, or clinical deterioration despite appropriate antimicrobial therapy [30]. Therefore, timing should be guided not only by the duration of hospitalization but also by the clinical trajectory and evidence of ongoing inflammation.

Treatment duration is generally short, most commonly ranging from 5 to 10 days in published pediatric studies [13,30,39,40,41]. Longer courses may occasionally be required in severe or refractory cases, particularly when inflammatory markers remain elevated, respiratory compromise persists, or radiological abnormalities continue to progress [11,14]. Although tapering is frequently used in clinical practice, especially after high-dose or prolonged therapy, there is no consensus regarding when tapering is necessary or how it should be performed [3,27,36]. Short courses may often be discontinued without tapering, whereas individualized tapering may be considered after pulse therapy, longer treatment duration, or in children at risk of adrenal suppression.

Overall, the choice of corticosteroid, dose, route, timing, and duration should be tailored to the clinical phenotype rather than applied uniformly to all children with CAP. Table 2 summarizes the main corticosteroid regimens reported in pediatric CAP, including their usual routes of administration, clinical contexts, and practical considerations.

### 3.5. Clinical Outcomes Associated with Corticosteroid Therapy

Several studies have assessed the effects of adjunctive systemic corticosteroids on short-term clinical outcomes in children with CAP. Overall, the available evidence suggests that potential benefits are most apparent in selected subgroups, particularly children with severe CAP, severe or refractory MPP, and complicated CAP characterized by marked inflammatory activity. However, reported outcomes vary substantially according to disease phenotype, timing of treatment, corticosteroid regimen, and study design.

The most consistently reported clinical benefit is a reduction in fever duration. In children with severe CAP or MPP, corticosteroid therapy has been associated with earlier defervescence, often within 24–48 h after treatment initiation, together with more rapid improvement in respiratory symptoms such as cough, tachypnea, dyspnea, and general clinical status [11,20,40,41]. This effect appears particularly relevant in patients with persistent fever despite appropriate antimicrobial therapy, in whom ongoing symptoms may reflect immune-mediated inflammation rather than uncontrolled microbial replication alone.

Adjunctive corticosteroid therapy has also been associated with a faster decline in inflammatory markers, including CRP, white blood cell count, and IL-6 [34,36]. These laboratory changes support the proposed anti-inflammatory effect of corticosteroids and may parallel clinical improvement. Nevertheless, reductions in biomarkers should be interpreted cautiously, as they do not necessarily translate into improved long-term outcomes and may be influenced by baseline disease severity, timing of sampling, and concomitant antimicrobial therapy.

Radiological improvement is another frequently reported outcome, especially in studies involving severe or refractory MPP. Several investigations have described faster resolution of pulmonary infiltrates among children treated with corticosteroids, with some studies reporting a lower frequency of persistent radiographic abnormalities during follow-up [20]. Early attenuation of inflammation may also reduce the risk of pulmonary sequelae such as atelectasis, airway obstruction, or bronchiectasis; however, evidence regarding long-term structural outcomes remains limited and requires further prospective evaluation [14,30].

Hospital length of stay (LOS) has been widely used as a clinically relevant endpoint. Randomized trials and meta-analyses have reported reductions in LOS ranging from approximately 1 to 5 days in selected pediatric populations receiving adjunctive corticosteroids [3,4,36]. However, findings from large retrospective studies have been less consistent, particularly when analyses include heterogeneous populations with variable disease severity, different etiologies, underlying comorbidities, or concomitant wheezing disorders [12,42]. These discrepancies suggest that corticosteroid benefit is unlikely to be uniform across all children with CAP and may depend strongly on patient phenotype.

In children with severe CAP, corticosteroids may also reduce inflammation-related complications and potentially limit progression toward acute respiratory distress syndrome (ARDS), although pediatric-specific evidence remains less robust than adult data [4]. In complicated CAP, particularly parapneumonic pleural effusion or empyema, adjunctive corticosteroids have been associated in some studies with more rapid clinical improvement, shorter hospitalization, and reduced need for invasive procedures such as chest drainage or surgical intervention [31,41]. These findings support a possible role for corticosteroids in selected inflammatory pleural complications, although they should not replace appropriate antimicrobial therapy or procedural management when indicated.

Beyond traditional clinical endpoints, a limited number of studies have examined patient- and caregiver-centered outcomes. Faster symptom resolution may reduce school absenteeism, caregiver work loss, healthcare utilization, and overall disease burden. Improvements in quality-of-life measures have been suggested in some investigations, but these outcomes remain underreported and should be incorporated into future studies [43].

Taken together, available data indicate that adjunctive corticosteroids may improve selected short-term outcomes in specific pediatric CAP phenotypes, particularly those characterized by severe inflammation, refractory disease, or complicated clinical courses. However, the heterogeneity of existing studies limits the ability to draw definitive conclusions. Table 3 summarizes representative studies evaluating clinical outcomes associated with corticosteroid therapy in pediatric CAP, highlighting differences in study design, population, intervention, outcomes assessed, and main findings [3,4,11,20,41,42].

The magnitude and reliability of the reported corticosteroid effects vary considerably according to study size and design. Several favorable findings derive from relatively small pediatric trials. For example, Nagy et al. randomized only 29 children with severe CAP and reported that adjunctive methylprednisolone reduced fever duration by approximately 2.5 days and hospital stay by 5.2 days compared with placebo [41]. Although these differences were clinically notable, the small sample size, single-center setting, and limited precision restrict the generalizability of the results. Similarly, Huang et al. included 106 children with MPP, with 53 assigned to corticosteroid treatment within 24 h of admission and 53 assigned to treatment after 72 h. Early treatment was associated with a shorter hospital stay (8 versus 10 days) and a shorter time to defervescence after corticosteroid initiation (0 versus 2 days) [20]. However, this trial compared early with delayed corticosteroid administration rather than corticosteroids with no corticosteroid therapy and therefore does not establish the overall efficacy of treatment.

Other studies were larger but observational and therefore more susceptible to confounding. Han et al. evaluated 56 children with microbiologically confirmed MPP who all received early corticosteroids; the absence of an untreated control group limits conclusions regarding treatment efficacy [11]. In contrast, a retrospective cohort of 885 hospitalized children with MPP found that low-dose corticosteroid treatment was associated with greater adjusted odds of fever lasting more than 3 days after admission, hospitalization longer than 8 days, and delayed CRP normalization, while no significant improvement in radiological recovery was identified [44]. These findings may partly reflect confounding by indication, because corticosteroids were more likely to be administered to children with more severe disease, but they demonstrate that favorable outcomes are not consistent across studies.

The largest pediatric analysis included 20,703 hospitalized children with CAP across 36 hospitals, of whom 7234 received adjunctive corticosteroids [42]. In children receiving bronchodilator therapy, corticosteroid use was associated with a shorter length of stay, whereas among children who did not receive bronchodilators, corticosteroid exposure was associated with a longer hospital stay and a greater risk of readmission. Although the large sample size increased statistical power, the retrospective design, reliance on administrative data, absence of detailed microbiological information, and possible misclassification of asthma or viral wheezing limit causal interpretation. The results suggest that wheezing or airway hyperreactivity may modify the observed association, but they do not demonstrate that corticosteroids directly improve pneumonia outcomes.

The 2026 systematic review included 22 studies comprising 75,353 children, but only seven studies provided data suitable for meta-analysis [3]. Sample sizes ranged from 12 to 20,703 participants, illustrating the marked imbalance between a few large observational datasets and numerous small clinical studies. In randomized studies, corticosteroids were associated with moderate reductions in length of stay (Cohen’s d = −0.59, 95% confidence interval [CI] −0.96 to −0.23) and fever duration (Cohen’s d = −0.54, 95% CI −0.83 to −0.26). These benefits were not consistently reproduced in observational studies, and substantial heterogeneity was present in corticosteroid regimen, dose, treatment duration, initiation timing, etiology, and disease severity [3]. Therefore, the pooled estimates should not be interpreted as evidence of a uniform treatment effect across pediatric CAP phenotypes.

Overall, numerical effect estimates should be interpreted in conjunction with study size, design, comparator, confidence intervals, and risk of bias. Large observational cohorts provide greater statistical precision but remain vulnerable to residual confounding and confounding by indication, whereas small randomized trials offer stronger causal inference but may produce imprecise or exaggerated estimates and have limited external validity. Accordingly, the available literature suggests possible short-term benefits in selected populations but does not establish a consistent effect on mortality, respiratory failure, intensive-care admission, readmission, or long-term pulmonary outcomes.

### 3.6. Safety Profile and Adverse Effects

Short courses of systemic corticosteroids are generally well tolerated in children, and serious adverse events are uncommon when treatment is appropriately selected and limited in duration [28]. Nevertheless, corticosteroids are not risk-free, and their safety profile must be carefully considered when evaluating their role as adjunctive therapy in pediatric pneumonia. This is particularly important because the expected benefit appears limited to selected phenotypes, whereas adverse effects may occur even after brief exposure.

Metabolic effects are among the most frequently reported complications. Hyperglycemia is commonly observed during systemic corticosteroid therapy, although it is usually transient and rarely requires specific treatment in otherwise healthy children [28,31,45]. Other short-term adverse events include vomiting, abdominal discomfort, increased appetite, weight gain, transient hypertension, sleep disturbance, and behavioral changes such as irritability, mood alteration, or agitation [45]. Although these events are generally reversible, they may affect tolerability and should be discussed when considering treatment.

The frequency and type of adverse effects may vary according to the corticosteroid formulation, dose, route of administration, and duration of therapy. Some evidence suggests that dexamethasone may be associated with fewer gastrointestinal adverse effects than prednisolone formulations, although direct comparisons in children with CAP remain limited [36]. Higher-dose regimens, pulse therapy, or prolonged courses may increase the likelihood of metabolic, cardiovascular, neurobehavioral, and endocrine effects, reinforcing the need for individualized dosing and close monitoring.

Suppression of the hypothalamic–pituitary–adrenal (HPA) axis has been documented after short-term corticosteroid exposure. In most cases, this suppression is biochemical and transient, with recovery of endogenous cortisol production occurring shortly after treatment discontinuation [45]. Clinically significant adrenal insufficiency appears uncommon after brief courses; however, the risk may increase with repeated courses, prolonged therapy, high cumulative doses, or concomitant use of other immunosuppressive agents.

Particular caution is required in viral CAP. In some severe viral respiratory infections, including those caused by adenovirus, influenza viruses, and SARS-CoV-2, corticosteroid therapy has been associated with concerns regarding delayed viral clearance, prolonged viral shedding, secondary infections, and poorer clinical outcomes [46,47,48]. In critically ill children admitted to pediatric intensive care units, corticosteroid exposure has also been associated with higher rates of nosocomial infection and worse outcomes in some observational studies [47]. This risk is likely to be influenced by corticosteroid dose, treatment duration, timing of administration, the causative pathogen, baseline immune status, and the use of concomitant immunosuppressive therapies. Particular caution is warranted in immunocompromised children and in patients receiving high-dose or prolonged regimens. However, causality is difficult to establish because corticosteroids are often administered to the most severely ill patients, creating a substantial risk of confounding by indication.

The timing of treatment may be critical. Corticosteroids administered during a phase dominated by uncontrolled viral replication may be more likely to impair clearance, whereas later administration in a hyperinflammatory phase may potentially reduce immune-mediated lung injury [46,47,48]. Nevertheless, reliable clinical or biomarker-based criteria for distinguishing these phases have not been validated in children. Therefore, systemic corticosteroids should not be routinely used in pediatric viral CAP unless there is a well-defined indication, such as concomitant asthma or wheezing, refractory shock, adrenal insufficiency, or another established inflammatory complication.

When corticosteroids are considered, clinicians should use the lowest effective dose for the shortest appropriate duration and ensure adequate antimicrobial therapy when bacterial coinfection is suspected or confirmed. Close monitoring is required for new or persistent fever, worsening respiratory status, hyperglycemia, leukocyte abnormalities, secondary infection, and delayed clinical recovery. Current evidence remains insufficient to quantify the risk of impaired pathogen clearance in pediatric CAP, and future prospective studies should incorporate serial microbiological testing, standardized definitions of secondary infection, and longer safety follow-up.

An additional clinical concern is that systemic corticosteroids may mask signs of inadequate antimicrobial response or evolving infectious complications by suppressing fever, cytokine production, leukocyte activation, and inflammatory biomarker concentrations [7,27,28,45]. Consequently, an early reduction in temperature, CRP, leukocyte count, or respiratory symptoms after corticosteroid initiation may reflect pharmacological suppression of inflammation rather than effective pathogen clearance. This effect could delay recognition of persistent infection, antimicrobial treatment failure, resistant organisms, coinfection, bacteremia, empyema, necrotizing pneumonia, or pulmonary abscess [10,31,46,47,48].

This risk is particularly relevant when corticosteroids are initiated before the causative pathogen has been adequately investigated or before the response to appropriate antimicrobial treatment can be evaluated. Because corticosteroids may reduce fever and inflammatory markers independently of microbiological resolution, treatment response should be assessed using multiple parameters, including oxygen requirement, respiratory effort, hemodynamic status, feeding and general condition, microbiological findings, radiological evolution, and the overall clinical trajectory [7,27,45]. Persistent hypoxemia, recurrent fever, worsening respiratory distress, or deterioration after an initial apparent improvement should prompt reassessment for treatment failure, inadequate antimicrobial coverage, resistant pathogens, secondary infection, or a complication requiring source control.

Accordingly, systemic corticosteroids should not substitute for appropriate pathogen-directed therapy, microbiological investigation, or close clinical monitoring. When corticosteroids are used during an active infection, clinicians should maintain a low threshold for repeat imaging, additional cultures or molecular testing, and reconsideration of antimicrobial therapy if the course is atypical or clinical improvement is not sustained [10,31,46,47,48]. The possibility that corticosteroids may obscure ongoing infection provides an additional reason to restrict their use to carefully selected patients in whom the anticipated benefit of controlling excessive inflammation outweighs the risk of delaying recognition of treatment failure or complications.

Long-term adverse effects are uncommon after a single short course, but they become more relevant with repeated exposure or prolonged treatment. Potential risks include growth suppression, reduced bone mineral density, osteopenia, and increased fracture risk, particularly in children receiving recurrent systemic corticosteroids or those with underlying chronic disease [27,36,49]. These concerns are especially important in pediatric practice, where cumulative exposure over time may have developmental consequences.

Overall, the safety profile of systemic corticosteroids in pediatric CAP supports a cautious, individualized approach. Treatment should be reserved for clinical situations in which the expected anti-inflammatory benefit is likely to outweigh potential harm, such as severe or refractory inflammatory phenotypes, rather than used routinely in uncomplicated CAP. Table 4 summarizes the principal adverse effects associated with systemic corticosteroid therapy in children, including their frequency, clinical relevance, and expected reversibility.

### 3.7. Corticosteroids in Community-Acquired Pneumonia with Wheezing Phenotype

A substantial proportion of children diagnosed with CAP present with concomitant wheezing or clinical features of airway hyperreactivity. This phenotype may reflect underlying asthma, viral-induced bronchospasm, transient airway inflammation, or previously undiagnosed reactive airway disease [42]. In these children, distinguishing infectious CAP from asthma exacerbation or viral wheezing illness can be difficult, particularly because symptoms such as cough, tachypnea, hypoxemia, and respiratory distress may overlap. This diagnostic uncertainty has important implications for interpreting the apparent benefit of corticosteroids.

Observational studies suggest that systemic corticosteroids are more frequently prescribed to children hospitalized with CAP who have wheezing or who receive bronchodilator therapy, especially inhaled beta-2 agonists [42]. In this subgroup, corticosteroid use has been associated with shorter hospital length of stay and more rapid clinical improvement [42]. However, these benefits may be primarily attributable to improvement in airway inflammation and bronchospasm rather than to a direct effect on the infectious pneumonic process. Therefore, wheezing should be considered a potential effect modifier when evaluating corticosteroid responsiveness in pediatric lower respiratory tract infections.

By contrast, in children with CAP without wheezing, asthma history, or evidence of airway obstruction, systemic corticosteroids have not shown consistent clinical benefit. Some studies have reported prolonged hospitalization, higher treatment failure, or increased readmission risk in non-wheezing children exposed to corticosteroids [42]. These findings suggest that the response to corticosteroids may depend more on the underlying clinical phenotype than on the diagnosis of CAP alone.

Misclassification between bacterial CAP, viral CAP, viral-induced wheezing, and asthma exacerbation may partly explain the heterogeneity observed across studies of corticosteroid therapy in pediatric CAP [42,43]. Future research should therefore stratify patients according to wheezing status, asthma history, bronchodilator response, viral testing, radiological findings, and inflammatory markers. Until more definitive evidence is available, systemic corticosteroids should not be used routinely for CAP solely on the basis of pneumonia diagnosis, but may be appropriate when there is a clear concomitant wheezing or asthma-exacerbation component.

### 3.8. Corticosteroids in Pneumocystis jirovecii Pneumonia

PJP represents a distinct form of infectious pneumonia that primarily affects immunocompromised patients. It occurs in children with HIV infection and, increasingly, in non-HIV immunosuppressed populations, including patients with hematologic malignancies, solid tumors, primary immunodeficiencies, transplant recipients, and those receiving immunosuppressive therapies [50,51].

The pathogenesis of PJP is characterized not only by fungal proliferation but also by a pronounced inflammatory response within the alveolar space, which contributes substantially to impaired gas exchange, hypoxemia, respiratory failure, and disease severity [51,52]. This inflammatory component provides the biological rationale for adjunctive systemic corticosteroids in selected patients, particularly when respiratory compromise is driven by immune-mediated alveolar injury.

The role of adjunctive corticosteroids is best established in HIV-associated moderate-to-severe PJP. Early randomized trials demonstrated that adding systemic corticosteroids to standard anti-Pneumocystis therapy reduced the risk of respiratory failure, need for mechanical ventilation, and mortality in patients with significant hypoxemia [53]. These findings were subsequently supported by systematic reviews and meta-analyses showing improved short-term survival and respiratory outcomes in HIV-associated PJP, especially when corticosteroids were started early in patients with moderate-to-severe disease [54].

The proposed mechanism of benefit is attenuation of the inflammatory response triggered by organism death after initiation of anti-*Pneumocystis* therapy. Without immunomodulation, this inflammatory surge may worsen alveolar damage, increase pulmonary permeability, and further impair gas exchange [54]. In this setting, corticosteroids act as a protective adjunct by limiting immune-mediated pulmonary injury during the acute treatment phase.

In contrast, the role of adjunctive corticosteroids in non-HIV PJP remains uncertain. Observational studies indicate that corticosteroids are commonly administered to patients with severe disease, but interpretation is limited by confounding by indication, since critically ill patients are more likely to receive immunomodulatory therapy [55]. Consequently, the absence of consistent survival benefit—or the association between corticosteroid exposure and worse outcomes in some studies—may reflect baseline disease severity rather than a direct harmful effect of corticosteroids.

Pediatric evidence in non-HIV PJP is particularly limited and is largely extrapolated from adult studies or derived from observational pediatric cohorts. Adult data have also been inconsistent. A recent multicenter randomized trial in HIV-negative immunocompromised patients did not demonstrate a statistically significant reduction in 28-day mortality with adjunctive corticosteroid therapy, although some secondary outcomes showed trends toward benefit [56]. These findings highlight the persistent uncertainty surrounding corticosteroid use in non-HIV PJP and underscore the need for better patient stratification.

Overall, systemic corticosteroids are an established adjunctive treatment for HIV-associated moderate-to-severe PJP with significant hypoxemia. By contrast, their role in non-HIV immunocompromised children remains uncertain and should be individualized according to disease severity, degree of hypoxemia, underlying immunosuppression, timing of antimicrobial therapy, and risk of secondary infection. In all cases, corticosteroids should be used as part of a comprehensive management strategy that includes prompt anti-*Pneumocystis* therapy, supportive respiratory care, and careful monitoring for complications.

### 3.9. Current Evidence, Limitations, and Future Directions

Despite growing interest in adjunctive corticosteroid therapy for pediatric CAP, the overall quality of the evidence remains limited. Much of the literature consists of retrospective analyses, single-center cohorts, and other observational studies, whereas adequately powered randomized controlled trials designed specifically for children remain scarce [10,36]. Consequently, current interpretations are constrained by methodological limitations and by the difficulty of separating true treatment effects from differences in disease severity, clinical indication, and patient characteristics.

A major limitation is the substantial clinical and methodological heterogeneity across studies. Published investigations differ in patient age, CAP etiology, microbiological confirmation, severity definitions, immune status, corticosteroid type, dose, route, timing, duration, concomitant therapy, and outcome measures [3,10,31]. Many studies also combine clinically distinct populations, including uncomplicated CAP, severe CAP, refractory MPP, viral CAP, wheezing-associated illness, asthma exacerbation, and complicated CAP. This overlap limits direct comparison, reduces generalizability, and may obscure whether observed benefits are related to treatment of pneumonia itself or to a coexisting corticosteroid-responsive condition such as airway hyperreactivity.

Diagnostic misclassification is another important source of uncertainty. Confirmation of *M. pneumoniae* and respiratory viral infections is difficult because no single test reliably establishes active lower respiratory tract infection early in the disease course [57,58]. For *M. pneumoniae*, a single serological result is insufficient: IgM may be absent early in infection, including in rapidly progressive disease, whereas prolonged IgM positivity after a previous episode may lead to false-positive interpretation. Polymerase chain reaction testing is also imperfect. False-negative results may occur because of sampling timing, specimen quality, prior antimicrobial exposure, or low organism burden, whereas positive upper-airway results may reflect carriage or prolonged detection rather than causative pneumonia. Similar limitations apply to respiratory viruses, for which nucleic acid detection may indicate active infection, presymptomatic infection, prolonged shedding, or incidental coinfection [58]. Microbiological findings should therefore be interpreted together with the clinical course, radiological features, inflammatory response, timing of sampling, and detection of alternative pathogens. When feasible, paired or repeated serology combined with serial molecular testing may improve diagnostic confidence for *M. pneumoniae*, although repeated serology is not a universal confirmatory standard for respiratory viral infections. Future studies should use predefined multimodal criteria and distinguish confirmed, probable, and possible infection.

The reported clinical effects of corticosteroids remain inconsistent. Some studies describe shorter fever duration, faster reductions in inflammatory markers, improved radiological resolution, or reduced hospital length of stay, particularly in severe or refractory MPP and selected complicated cases [3,11,30,48]. Other observational studies have found no benefit or have reported longer hospitalization, greater treatment failure, or increased readmission, especially in unselected populations or in children without wheezing [10,12]. These conflicting findings are likely influenced by differences in patient selection, disease phenotype, timing and dose of corticosteroids, concomitant therapy, and outcome definitions.

Confounding by indication is particularly important. Children receiving corticosteroids are often more severely ill, have persistent fever or respiratory deterioration, or are considered more likely to have a hyperinflammatory phenotype. Worse outcomes among treated patients may therefore reflect the severity that prompted treatment rather than corticosteroid-related harm. Conversely, apparent benefits may be influenced by preferential treatment of children thought most likely to respond. Residual confounding may persist even after multivariable adjustment or propensity-score methods, especially in retrospective cohorts and case–control studies.

Differences in patient composition further complicate interpretation. Adult CAP, influenza, and COVID-19 studies often include older patients with diabetes, hypertension, cardiovascular disease, chronic lung disease, obesity, malignancy, or immunodeficiency, all of which may independently affect prognosis and corticosteroid exposure. Pediatric studies may involve fewer age-related comorbidities, but they remain vulnerable to confounding from age-dependent immune maturation, prematurity, congenital disease, asthma or wheezing, nutritional status, vaccination history, immune deficiency, pathogen distribution, previous antimicrobial therapy, and access to care. Adult findings should therefore not be extrapolated directly to children without careful consideration of differences in host biology and baseline risk.

Important uncertainties also remain regarding patient selection. CRP, LDH, ferritin, IL-6, and IL-18 may provide prognostic information or support monitoring of inflammatory activity, but none has been sufficiently validated to independently predict corticosteroid responsiveness or guide treatment initiation [11,14,32,33,34,35]. Proposed thresholds derive mainly from retrospective, single-center, or phenotype-specific studies and lack adequate external validation. Similarly, the optimal corticosteroid regimen remains undefined. Available studies differ widely in the use of methylprednisolone, pulse methylprednisolone, prednisone or prednisolone, and dexamethasone, as well as in dose, route, duration, and tapering strategy [3,27,36]. The therapeutic window is also uncertain: some patients may benefit from prompt treatment after rapid inflammatory deterioration, whereas others may require immunomodulation only after persistent or refractory disease becomes evident [11,20,30,40].

Current pediatric guidelines reflect this uncertainty. The 2024 World Health Organization guideline for childhood pneumonia emphasizes clinical classification, antimicrobial therapy, oxygen assessment, supportive care, and management of mortality risk factors, but does not recommend systemic corticosteroids as routine adjunctive therapy for uncomplicated CAP [59]. Similarly, the 2026 Pediatric Infectious Diseases Society/Infectious Diseases Society of America guideline does not establish a general indication for adjunctive corticosteroids in uncomplicated pediatric CAP [60].

Corticosteroids may nevertheless be appropriate when a child with CAP has a separate, recognized corticosteroid-responsive condition, such as an asthma exacerbation, clinically significant airway obstruction, adrenal insufficiency, or selected forms of critical inflammatory illness. In such cases, they are prescribed for the associated condition rather than as routine pneumonia treatment. Evidence is also insufficient to support a uniform corticosteroid recommendation in pediatric sepsis or septic shock, and current Society of Critical Care Medicine guidance does not endorse routine use in pediatric sepsis [61].

Pathogen-specific exceptions should be considered separately from general CAP. The World Health Organization recommends systemic corticosteroids for severe or critical COVID-19 with hypoxemic respiratory disease but advises against routine use in non-severe COVID-19 because benefit has not been demonstrated and harm remains possible [62,63]. This recommendation is based predominantly on adult evidence and should therefore be applied to children with attention to pediatric disease severity, comorbidities, and the limited pediatric trial data.

No major pediatric CAP guideline currently recommends initiating corticosteroids solely on the basis of CRP, LDH, ferritin, IL-6, or IL-18 concentrations, nor do guidelines provide standardized recommendations regarding corticosteroid choice, dose, route, initiation threshold, duration, or tapering in severe or refractory CAP. Use in severe CAP, refractory MPP, or inflammatory pleural complications therefore remains individualized and is based on limited randomized evidence, observational studies, local practice, and specialist judgment. The absence of a guideline recommendation should not be interpreted as proof of either efficacy or ineffectiveness; rather, it reflects the lack of sufficiently consistent pediatric data.

The current evidence base is also limited by its emphasis on short-term outcomes. Most studies focus on defervescence, inflammatory-marker changes, radiological improvement, or length of stay, whereas evidence on mortality, mechanical ventilation, recurrence, long-term pulmonary function, bronchiectasis, persistent radiological abnormalities, quality of life, and cumulative corticosteroid toxicity remains insufficient. Safety data are further constrained by small sample sizes, short follow-up, and inconsistent adverse-event reporting.

Overall, the available literature does not establish a uniform net benefit of systemic corticosteroids in pediatric CAP and does not support routine use in unselected children. Their most plausible role remains in carefully selected patients with severe inflammation, refractory disease, pleural complications, or a separate corticosteroid-responsive condition, but these indications require confirmation.

Future research should prioritize large, multicenter pediatric randomized controlled trials using standardized definitions of CAP severity, etiology, inflammatory phenotype, treatment response, and adverse events. Studies should stratify or adjust for age, immune status, comorbidities, wheezing, pathogen, symptom duration, timing of corticosteroid initiation, and concomitant therapy. They should also incorporate validated diagnostic criteria, biomarker-based subgroup analyses, harmonized corticosteroid regimens, and long-term follow-up. Such trials are needed to determine whether corticosteroid effects are attributable to treatment rather than to differences in study populations and to define which children, if any, derive a clinically meaningful net benefit.

## 4. Toward a Phenotype-Driven Use of Corticosteroids in Pediatric Community-Acquired Pneumonia

The biological and clinical diversity of pediatric CAP complicates attempts to define a uniform role for systemic corticosteroids. Respiratory viruses, typical bacteria, and *M. pneumoniae* differ in cellular tropism, replication strategy, virulence factors, tissue invasion, and activation of host immune pathways [5,14,16,19]. Nevertheless, these pathogens may produce overlapping clinical syndromes through convergent mechanisms, including epithelial and endothelial injury, disruption of the alveolar–capillary barrier, inflammatory-cell recruitment, vascular leakage, and impaired gas exchange [5,16,17,18]. The potential effect of corticosteroids therefore cannot be inferred from the diagnostic label alone.

Respiratory viruses may cause epithelial injury through direct cytopathic effects, disruption of barrier integrity, induction of cell death, and activation of antiviral interferon pathways [5,46,47,48]. During the early replicative phase, inflammatory responses are essential for viral control; however, persistent or excessive activation may subsequently amplify pulmonary injury. Corticosteroid administration during uncontrolled viral replication could therefore impair antiviral immunity or delay viral clearance, whereas treatment during a later phase dominated by dysregulated inflammation might reduce host-mediated tissue damage [46,47,48]. Routine clinical tests do not reliably distinguish these phases.

Typical bacterial pathogens may induce lung injury through proliferation, release of toxins and cell-wall or membrane components, and activation of predominantly neutrophilic inflammatory responses [5,16,17,18]. In this setting, severity may depend on microbial burden, the anatomical extent of infection, persistence of pathogen-derived inflammatory stimuli, timing and effectiveness of antimicrobial therapy, and the host’s capacity to contain infection. Corticosteroid treatment before adequate bacterial control could be harmful, whereas adjunctive treatment may be biologically plausible in selected patients with persistent inflammation despite appropriate antimicrobial therapy.

The pathophysiology of MPP is similarly multifactorial. Attachment to respiratory epithelial cells, impairment of ciliary function, oxidative and membrane injury, and pathogen-derived inflammatory factors may interact with an exaggerated host response [14,16,19,20]. This interaction may explain why most infections remain mild and self-limited, while a minority of children develop persistent fever, extensive radiological abnormalities, hypoxemia, extrapulmonary manifestations, or refractory disease [14,19,30]. Although this provides a rationale for corticosteroids in selected severe or refractory cases, *M. pneumoniae* detection alone does not identify a corticosteroid-responsive condition.

Marked interindividual variability is evident across influenza, COVID-19, and MPP. Patients infected by the same organism may have substantially different clinical presentations, trajectories, and outcomes [14,19,30,46,47,48]. Most children recover without major complications, but severe pneumonia, rapidly progressive respiratory failure, or ARDS may occur even in previously healthy and immunocompetent individuals [14,20,30,46,47]. Disease expression probably reflects the interaction among pathogen burden and virulence, anatomical distribution, timing of clearance, previous immune exposure, age, host susceptibility, and the magnitude and regulation of the inflammatory response [5,14,16,19].

These observations also clarify the relationship between mild and severe disease. Mild and severe pneumonia need not represent fundamentally different immunopathogenic processes. Rather, severity may reflect differences in the magnitude, duration, and localization of the infectious or inflammatory stimulus relative to the host’s capacity to control it without causing collateral tissue injury. Mild infections are generally self-limited because pathogen-derived stimuli are contained and inflammation remains proportionate. In severe disease, greater microbial burden, delayed clearance, extensive cellular injury, impaired host regulation, or inflammatory amplification may shift this balance toward progressive pulmonary damage.

ARDS represents an extreme manifestation of these converging processes. Infectious and non-infectious insults—including viral or bacterial infection, aspiration, trauma, toxic inhalation, burns, pancreatitis, and autoimmune disease—may activate shared pathways of epithelial and endothelial injury, alveolar–capillary permeability, leukocyte recruitment, and diffuse alveolar damage [5]. The initiating insult varies, but the final pathways of respiratory failure may overlap. Therapeutic response may therefore depend more on the dominant mechanism and temporal phase of injury than on etiology alone.

Accordingly, the term “corticosteroid-responsive phenotype” should be used cautiously. Severe pneumonia, respiratory compromise, refractory disease, pleural inflammation, and wheezing-associated presentations are pragmatic clinical categories rather than validated biological phenotypes. They may serve as imperfect indicators that inflammation contributes to disease expression, but they do not demonstrate a common mechanism or independently predict corticosteroid benefit. A more precise objective is to identify mechanistically defined endotypes characterized by pathogen burden, epithelial and endothelial injury, immune activation, disease phase, and host-response capacity.

This distinction is particularly important in wheezing-associated CAP. In the large observational study by Weiss et al., corticosteroid exposure was associated with shorter hospitalization among children receiving bronchodilators, but with longer hospitalization and increased readmission among those without bronchodilator use [42]. The apparent benefit in wheezing children may primarily reflect treatment of bronchospasm and airway inflammation rather than a direct effect on pneumonia. Wheezing status, asthma history, and bronchodilator use should therefore be treated as important effect modifiers rather than as evidence that CAP itself is corticosteroid responsive.

The evidence in MPP is also conflicting. Selected studies have associated early corticosteroid administration with faster defervescence and clinical or radiological improvement, particularly in severe or refractory disease [11,20,30,35]. Conversely, other observational analyses have reported prolonged fever, hypoxemia, more extensive radiological involvement, or no clear benefit [44]. Differences in baseline severity, microbiological criteria, timing, dose, concomitant treatment, and confounding by indication probably contribute to these inconsistencies. Because the most severely affected children are more likely to receive corticosteroids, unfavorable outcomes may reflect the clinical indication for treatment rather than a direct harmful effect. Conversely, apparent benefits may arise from preferential selection of patients considered likely to respond.

The limitations identified by the Cochrane review remain relevant: pediatric evidence is insufficient to define a consistent treatment effect or establish the optimal drug, dose, timing, duration, and target population [10]. Although the 2026 pediatric systematic review provides an updated synthesis, the underlying studies remain heterogeneous in design, sample size, disease phenotype, corticosteroid regimen, and outcome definition [3]. Adult findings should not be extrapolated directly because children differ in immune maturation, pathogen distribution, comorbidity burden, and pharmacological response [3,10].

Timing may be especially important in rapidly progressive disease. Theoretically, immunomodulation may be most effective after dysregulated inflammation has begun to drive pulmonary injury but before diffuse alveolar damage and established ARDS become advanced or irreversible [5,7,10]. The clearest clinical evidence for this principle derives from severe COVID-19. In the RECOVERY trial, dexamethasone reduced 28-day mortality among hospitalized patients requiring oxygen or invasive mechanical ventilation, but not among patients who did not require respiratory support [64]. This evidence informed the World Health Organization recommendation to use systemic corticosteroids in severe or critical COVID-19 and to avoid routine administration in non-severe disease [62].

The COVID-19 experience supports prompt treatment after objective evidence of severe respiratory involvement, rather than indiscriminate administration at the onset of infection. In this context, “early” should refer to initiation after the development of hypoxemia, escalating oxygen requirements, rapid radiological progression, or other evidence of severe inflammatory lung disease. It should not imply prophylactic or preemptive treatment of mild, uncomplicated infection, in which spontaneous recovery is expected and suppression of pathogen clearance may be harmful [46,47,48].

Whether the same principle applies to severe influenza or MPP remains uncertain. In influenza-associated pneumonia and ARDS, observational evidence has been inconsistent and is affected by confounding by indication, secondary infection, and concerns regarding delayed viral clearance [46]. In severe or refractory MPP, early corticosteroid administration has been associated with improved short-term outcomes in selected pediatric studies [11,20,30,35], but heterogeneous severity definitions, regimens, and comparators preclude a universal recommendation for treatment before ARDS develops.

Evidence for intravenous immunoglobulin (IVIG) is substantially weaker. Although IVIG has immunomodulatory properties and has been used in selected severe or extrapulmonary infections, its role in pediatric CAP has not been established through adequately powered controlled trials. It should therefore not be regarded as standard preemptive treatment outside established indications or research protocols. A specific reference on IVIG in severe MPP or viral pneumonia should be added if this statement is retained.

Overall, current evidence does not support the assumption that corticosteroid efficacy is determined simply by CAP etiology or a descriptive clinical phenotype. Benefit is most biologically plausible when dysregulated inflammation has become a major driver of deterioration despite adequate pathogen-directed treatment. Harm is more plausible when pathogen replication remains uncontrolled, when the immune response is still required for clearance, or when the expected course is self-limited [10,14,46,47,48]. No routinely available clinical variable or individual biomarker reliably distinguishes these biological states.

Safety remains central to this assessment. Short courses are generally tolerated, but metabolic, gastrointestinal, neurobehavioral, and sleep-related adverse effects are common, while immunosuppression, secondary infection, delayed pathogen clearance, and masking of anti-infective treatment failure are clinically important concerns [27,45,46,47,48]. These risks are particularly relevant in uncomplicated CAP, in which the probability of benefit is low.

The principal research priority is therefore to move from broad diagnostic labels toward mechanistically defined endotypes. Future multicenter pediatric trials should combine quantitative pathogen assessment with longitudinal evaluation of inflammatory mediators, immune-cell activation, epithelial and endothelial injury, host transcriptomics or proteomics, imaging, and clinically meaningful outcomes. Studies should stratify patients by disease phase, etiology, severity, wheezing status, immune status, and timing of corticosteroid initiation. Long-term outcomes—including recurrent disease, pulmonary function, structural sequelae, quality of life, and cumulative toxicity—should be assessed alongside short-term endpoints such as fever duration and hospital stay.

Until such evidence is available, systemic corticosteroids should not be regarded as universally beneficial adjunctive therapy for pediatric CAP. Their use should remain individualized and limited to established corticosteroid-responsive conditions or carefully selected patients with severe or refractory disease in whom inflammation is strongly suspected to be a major contributor to ongoing lung injury.

## 5. Conclusions

Systemic corticosteroids are a biologically plausible adjunctive therapy in pediatric CAP because of their capacity to attenuate excessive host inflammatory responses and potentially limit inflammation-mediated lung injury. However, current evidence does not support their routine use in all children with CAP [3,4,10].

The available data suggest that corticosteroids may offer benefit in carefully selected clinical scenarios, particularly in children with severe CAP, refractory or severe MPP, complicated pneumonia with prominent inflammatory involvement, or pneumonia associated with airway hyperreactivity or asthma exacerbation. In these phenotypes, corticosteroids may contribute to faster fever resolution, improvement in inflammatory markers, shorter hospitalization, or more rapid clinical stabilization. Conversely, in uncomplicated CAP or unselected pediatric populations, the expected benefit appears limited and may be outweighed by potential risks, including metabolic, behavioral, gastrointestinal, and infection-related adverse effects [37,52].

The current evidence base remains limited by substantial heterogeneity in study design, patient populations, pneumonia etiologies, severity definitions, corticosteroid regimens, timing of administration, and outcome measures. High-quality pediatric randomized controlled trials are scarce, and uncertainty persists regarding optimal patient selection, biomarker-guided risk stratification, drug choice, dosage, duration, and tapering strategies.

Several limitations should be considered when interpreting the evidence summarized in this review. The current understanding of pediatric CAP remains incomplete because its etiology is often heterogeneous or only partially defined, and the relative contributions of pathogen-related injury, host inflammatory responses, and patient-specific susceptibility may vary over the course of illness. Consequently, several mechanistic interpretations, proposed biomarkers, and phenotype-based indications for corticosteroid therapy discussed in this review should be regarded as provisional rather than definitive. The available evidence supports biologically plausible hypotheses and identifies clinical patterns that may guide future investigation, but it does not establish a single unifying pathophysiological model or universally applicable treatment strategy. Future prospective studies integrating microbiological diagnosis, longitudinal immune profiling, measures of tissue injury, disease severity, treatment timing, and clinically meaningful outcomes may confirm, refine, or challenge the concepts presented here. Accordingly, the conclusions of this review should be interpreted as a framework for hypothesis generation and individualized clinical assessment rather than as proof of causality or final therapeutic guidance.

Corticosteroid therapy in pediatric CAP should therefore be regarded as a targeted, phenotype-driven intervention rather than a routine adjunctive treatment. Until more robust evidence becomes available, its use should be individualized according to disease severity, inflammatory burden, underlying etiology, wheezing status, clinical trajectory, and risk–benefit assessment. Well-designed prospective multicenter studies are urgently needed to define precise indications, standardize therapeutic regimens, assess long-term safety, and establish evidence-based pediatric guidelines for corticosteroid use in pneumonia.

## Figures and Tables

**Table 1:** Biomarkers for Patient Selection and Risk Stratification in Pediatric Community-Acquired Pneumonia.

Biomarker	Pathophysiological Role	Clinical Association	Proposed Threshold	Clinical Utility
C-reactive protein (CRP)	Systemic inflammation marker	Disease severity in CAP	≥44 mg/L	Supports severity assessment
Lactate dehydrogenase (LDH)	Tissue damage/lung injury	Severe MPP, extensive infiltration	≥590 IU/L	Marker of pulmonary injury and severity
Ferritin	Hyperinflammatory response	Severe systemic inflammation	≥411 ng/L	Possible indication for high-dose therapy
Interleukin-6 (IL-6)	Pro-inflammatory cytokine	Severe inflammatory response	No validated cutoff	Monitoring disease activity
Interleukin-18 (IL-18)	Innate immune activation	Severe pneumonia phenotypes	Not validated	Research use

Abbreviations: CAP, community-acquired pneumonia; MPP, *Mycoplasma pneumoniae* pneumonia.

**Table 2:** Corticosteroid Regimens Used in Pediatric Community-Acquired Pneumonia.

Drug	Dose	Route	Typical Indication	Notes
Methylprednisolone	2–30 mg/kg/day	Intravenous	Severe CAP/MPP	Dose-dependent anti-inflammatory effect
Pulse methylprednisolone	10–30 mg/kg/day	Intravenous	Refractory MPP/hyperinflammatory states	Rapid clinical response
Prednisone/Prednisolone	1–2 mg/kg/day	Oral	Mild–moderate CAP	Step-down therapy
Dexamethasone	0.2–1 mg/kg/day	IV or oral	Empyema/parapneumonic effusion	Associated with reduced LOS
Duration of therapy	5–10 days	—	Most CAP cases	No standardized protocol

Abbreviations: CAP, community-acquired pneumonia; LOS, hospital length of stay; MPP, *Mycoplasma pneumoniae* pneumonia.

**Table 3:** Clinical Outcomes Associated with Corticosteroid Therapy in Pediatric Community-Acquired Pneumonia.

Study	Design	Population	Intervention	Primary Outcomes	Main Findings
Han et al., 2021 [11]	Retrospective cohort	Pediatric MPP	Early methylprednisolone	Fever duration, LOS	Reduced fever duration and hospital stay
Huang et al., 2014 [20]	Randomized controlled trial	Severe MPP	Corticosteroids vs. standard care	Radiologic resolution	Faster radiographic improvement
Nagy et al., 2013 [41]	Randomized controlled trial	Severe CAP	Methylprednisolone	Fever duration	~2.5-day reduction in fever duration
Wan et al., 2016 [4]	Systematic review and meta-analysis	CAP (adults + pediatric subgroup)	Adjunctive corticosteroids	Mortality, ARDS, LOS	Reduced complications and LOS in selected cases
Weiss et al., 2011 [42]	National retrospective study	Hospitalized CAP patients	Systemic corticosteroids	LOS, healthcare costs	Increased LOS in unselected population
Geropeppa et al., 2026 [3]	Systematic review and meta-analysis	Pediatric CAP	Corticosteroids adjunctive therapy	Clinical outcomes	Benefit mainly in selected phenotypes

Abbreviations: ARDS, acute respiratory distress syndrome; CAP, community-acquired pneumonia; LOS, hospital length of stay; MPP, Myco-plasma pneumoniae pneumonia.

**Table 4:** Adverse Effects of Systemic Corticosteroids in Pediatric Community-Acquired Pneumonia.

System	Adverse Effect	Frequency (Reported)	Clinical Relevance	Reversibility
Metabolic	Hyperglycemia	Common	Usually mild, transient	Yes
Gastrointestinal	Vomiting	~5%	May limit adherence	Yes
Neurobehavioral	Irritability, sleep disturbance	4–10%	Common in children	Yes
Cardiovascular	Transient hypertension	Variable	Usually mild	Yes
Immune system	Secondary infections	Rare (~1%)	Clinically relevant	Variable
Endocrine	HPA axis suppression	High biochemical incidence	Rare clinical insufficiency	Yes
Growth/bone	Growth suppression (recurrent use)	Long-term risk	Cumulative exposure dependent	Partially reversible

Abbreviation: HPA, hypothalamic–pituitary–adrenal.

## Data Availability

No new data were created or analyzed in this study. Data sharing is not applicable to this article.
